# Dendritic cell vaccines improve the glioma microenvironment: Influence, challenges, and future directions

**DOI:** 10.1002/cam4.5511

**Published:** 2022-12-04

**Authors:** Jing Zhou, Luohong Li, Minqi Jia, Qianjin Liao, Guiping Peng, Gengqiu Luo, Yanhong Zhou

**Affiliations:** ^1^ NHC Key Laboratory of Carcinogenesis, Hunan Cancer Hospital and The Affiliated Cancer Hospital of Xiangya School of Medicine Central South University Changsha Hunan China; ^2^ Cancer Research Institute, Basic School of Medicine Central South University Changsha Hunan China; ^3^ Hunan Key Laboratory of Cancer Metabolism, Hunan Cancer Hospital and the Affiliated Cancer Hospital of Xiangya School of Medicine Central South University Changsha Hunan China; ^4^ Department of Radiation Oncology Peking University Cancer Hospital & Institute Beijing China; ^5^ Xiangya School of Medicine Central South University Changsha China; ^6^ Department of Pathology, Xiangya Hospital, Basic School of Medicine Central South University Changsha Hunan China

**Keywords:** dendritic cells, glioma, treatment, tumor microenvironment, vaccine

## Abstract

**Introduction:**

Gliomas, especially the glioblastomas, are one of the most aggressive intracranial tumors with poor prognosis. This might be explained by the heterogeneity of tumor cells and the inhibitory immunological microenvironment. Dendritic cells (DCs), as the most potent in vivo functional antigen‐presenting cells, link innate immunity with adaptive immunity. However, their function is suppressed in gliomas. Therefore, overcoming the dysfunction of DCs in the TME might be critical to treat gliomas.

**Method:**

In this paper we proposed the specificity of the glioma microenvironment, analyzed the pathways leading to the dysfunction of DCs in tumor microenvironment of patients with glioma, summarized influence of DC‐based immunotherapy on the tumor microenvironment and proposed new development directions and possible challenges of DC vaccines.

**Result:**

DC vaccines can improve the immunosuppressive microenvironment of glioma patients. It will bring good treatment prospects to patients. We also proposed new development directions and possible challenges of DC vaccines, thus providing an integrated understanding of efficacy on DC vaccines for glioma treatment.

## Graphical abstract

Dendritic cell vaccines improve the glioma microenvironment. This review provides an integrated understanding of efficacy on DC vaccines for glioma treatment.

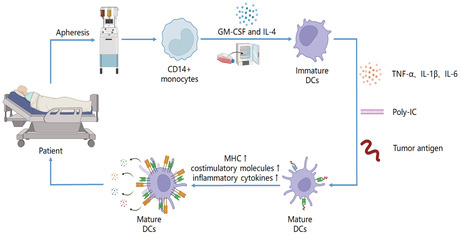

## 1 INTRODUCTION

Glioma is the most common primary intracranial tumor with a 5‐year survival rate of <10%.^1^ According to the WHO grade 2016, it can be divided into four grades, and the fourth grade is also known as glioblastoma (GBM).^2^ Many kinds of cancer medicine have been invented over the past few decades. However, few of them were approved by the US Food and Drug Administration (FDA) to treat gliomas.^3^ The special inhibitory tumor microenvironment (TME) might be one of the important reason for the limited efficacy of current drugs.^4^ On the one hand, central nervous system (CNS) has been recognized as the immunological privilege site, in which the blood–brain barrier (BBB) prevents immune cells from infiltrating the CNS.^5^ On the other hand, some specific constituent cells (astrocytes, microglia, and neurons, etc.) in the CNS can aggravate the glioma proliferation, inhibit immune cells like dendritic cells and T cells, thus creating a more severe immunosuppressive microenvironment in patients with glioma.

Dendritic cells (DCs), as the most potent in vivo functional antigen‐presenting cells, link innate immunity with adaptive immunity.^6^ However, their function is suppressed in gliomas.^7^ Therefore, overcoming the dysfunction of DCs in the TME might be critical to treat gliomas.^8^ DC vaccines can play a therapeutic role by upregulating major histocompatibility complex (MHC), co‐stimulator expression and the levels of cytokines or chemokines,^9^ which increase activated T cells, promote cell migration, and initiate the adaptive immune reaction, thus improving TME of patients with glioma.^10^ DC vaccines comprise DCs sampled from the patient who treats in vitro and then induce an immunological reaction against the tumor when reintroduces them into patients. Because the sipuleucel‐T was formally approved by the FDA for metastatic prostate cancer and inclusion in the clinical protocol in 2010,^11^ the FDA has approved the successful use of autologous DC‐based cancer vaccines for other tumors like melanoma. However, because of the tumor heterogeneity of gliomas and their special TME, gliomas are highly prone to antigen loss evasion,^12^ which limits the efficacy of single DC‐based cancer vaccines. Therefore, further optimization of DC vaccines is of great significance to improve their efficacy and patients' survival.^13^ In this review, we analyzed several pathways causing DC dysfunction in immune microenvironment of glioma patients, summarized influence of DC‐based immunotherapy on the tumor microenvironment and proposed possible challenges of DC vaccines and new development directions.

## 2 THE PARTICULARITY OF THE GLIOMA MICROENVIRONMENT

Compared with the tumors in other parts of the body, the glioma microenvironment is unique in the special structure of CNS and its particular cell types. CNS has been considered as an immune privileged site because of the presence of blood–brain barrier (BBB) for many years.^9^ BBB consists of pericytes, astrocyte foot processes, extracellular matrix, and vascular endothelial cells, which protect the brain from pathogenic microorganisms, and make it difficult for drugs and peripheral immune cells to penetrate into the CNS as well, thus favoring tumor infiltration and growth.^14^ However, recent studies have shown that immunization in the CNS is considered “unique” rather than “privileged”,^15^ and it may have lymphatic system where immune cells can enter the arachnoid villi into the central venous sinus or into the lymphatic duct via the sieve plate and drain into the deep cervical lymph nodes,^16^ which achieves the participation of systemic immune system against glioma antigens.^17^ Therefore, when inflammation occurs, microglia recognize specific antigens and present them to activated lymphocytes via the glial lymphoid pathway,^18^ after which large numbers of immune cells readily penetrate the BBB, inducing a strong inflammatory response and a subsequent immune response. Nonetheless, both these responses must be tightly regulated,^19^ and the impaired BBB upregulates the expression of program death ligand 1/2 (PD‐L1/PD‐L2) to inhibit the effector T cell activation, thus inhibiting the adaptive immune response in glioma patients.

In the CNS of patients with glioma, there are some unique constituent cell types, including neurons, astrocytes, and microglia, which can aggravate the immunosuppression of the glioma microenvironment through their physical or chemical effects.^20^ On the one hand, the astrocytes form a scar surrounding the glioma cells through their activation, thus physically “clearing” the T lymphocytes on the glioma cells to form a cold tumor phenotype.^21^ On the other hand, astrocytes are activated under the co‐drive driven by microglia and secrete interleukin 10 (IL‐10) and CSF through the JAK/STAT pathway to inhibit T cell activation.^22^ Microglia cells can upregulate GM‐CSF and stromal derived factor‐1 (SDF‐1), which aggravate the growth and invasion of glioma cells.^23^ Besides, neurons can secrete inhibitory cytokines vascular endothelial growth factor (VEGF) and express CD200, inhibiting T cells to initiate the immune response. Neurons can also release the mitogen neuroligin‐3 (NLGN3) and promote the glioma cell proliferation via the PI3K‐mTOR signaling pathway,^24^ which is related to survival in patients with high‐grade glioma. Therefore, although blood‐derived immune cells can infiltrate into the CNS through the BBB and meningeal lymphatic vessels, the specificity of the glioma microenvironment makes immune cells' function suppressed, such as DCs, T cells, and NK cells.^25^

## 3 THE GLIOMA MICROENVIRONMENT CAN CAUSE DYSFUNCTION OF DCS

### 3.1 DCs in the glioma

Normally, peripheral circulating DCs travel through central lymph duct and arrive at the vascular‐rich compartments (e.g., chorioid), so they are hardly present in the brain parenchyma.^26^ DCs originate from bone marrow hematopoietic stem cell (BM‐HSCs) and produce myeloid dendritic cells (mDCs) and plasmacytoid dendritic cells (pDCs).^27^ DCs can also originate from monocytes and produce MoDCs. Depending on the different phenotypes, the mDCs are mainly divided into the cDC1 (CD141+) and cDC2 (CD1c+) subgroups.^28^ cDC1s can express Cleca9A, XCR1 and CD141, which are related to perform cross‐presenting antigen to CD8+ T cells.^29^ cDC2s can express CD1c+ and CD172a. The cDC2s can stimulate CD4+ T cell differentiation and participate in the immune response.^30^ Furthermore, slanDC is a non‐classical subset of mDCs and shares several features with monocytes, particularly their pro‐inflammatory properties and association with inflammatory diseases.^31^

In patients with glioma, the abundance and phenotype of the DC subtypes have changed. In 2019, Adhikaree J et al. first examined abundance of circulating DC and its associated phenotypic in GBM patients. They found that GBM patients had a decrease in circulating cDC2s (CD1c+), while the slanDC subset was unaffected.^25^ Furthermore, the circulating cDC2s in the GBM patients show a significant reduction in HLA‐DR and CD86 expression,^32^ which represents an immature DC phenotype that can lead to T cell tolerance. Therefore, DCs are in the inhibitory or immature state, which may be related to the severe TME.^33^ Glioma cells have intrinsic resistance to DCs and other cells with immune surveillance in the CNS.^34^ The hypoxic environment will also damage the BBB and affect tumor cells metabolism, inhibiting DCs from exerting their anti‐tumor response, leading to cancer proliferation and immune escape.^35^

### 3.2 Intrinsic resistance effect of glioma cells to DCs

Differentiation of DCs is inhibited by cytokines secreted by glioma cells,^36^ such as IL‐10, IL‐6, prostaglandin E2 (PGE2) and VEGF. PGE2 produced by tumor cells promotes IL‐10 production by DCs, thereby inhibiting effector T cell responses.^37^ IL‐6 can reduce its MHC and CD80/86 expression by activating the STAT3 pathway to interfere with DC maturation.^32^

Certain products of glioma cells have been associated with DC dysfunction, including 2‐hydroxyglutarate (2‐HG), fibrinogen‐like protein 2 (FGL2),^38^ Nrf2, and thymic stromal lymphopoietin (TSLP) dehydrogenase. In the glioma patients with isocitrate dehydrogenase (IDH) mutation, β‐ketoglutarate is converted to D‐2‐HG and the latter appears to drive extensive epigenetic changes that alter cell differentiation and possibly promote tumorigenesis.^39^ D‐2‐HG can lead to specifically educated, dysfunctional DCs by reprogramming of the lipopolysaccharide (LPS)‐induced metabolism,^38^, ^40^ promoting oxidative phosphorylation, inhibiting glycolysis, and inhibiting p34/IL‐12A and p35/IL‐12B expression,^41^ thus reducing IL‐12 and promoting immune escape from tumor cells.^42^ Glioma cells can induce Nrf overexpression in the DCs, which in turn leads to the inhibition of DC maturation and reduced effector T cell activation.^43^ Inhibition of the Nrf2 pathway rescued the maturation of both CD80+ and CD86+ DC in the conditioned media of glioma cells. TSLP can upregulate OX40 receptor expression on the DC surface, prompting the release of IL‐4 and IL‐13 by Th2 cells to induce immunosuppression.^44^

**FIGURE 1:**
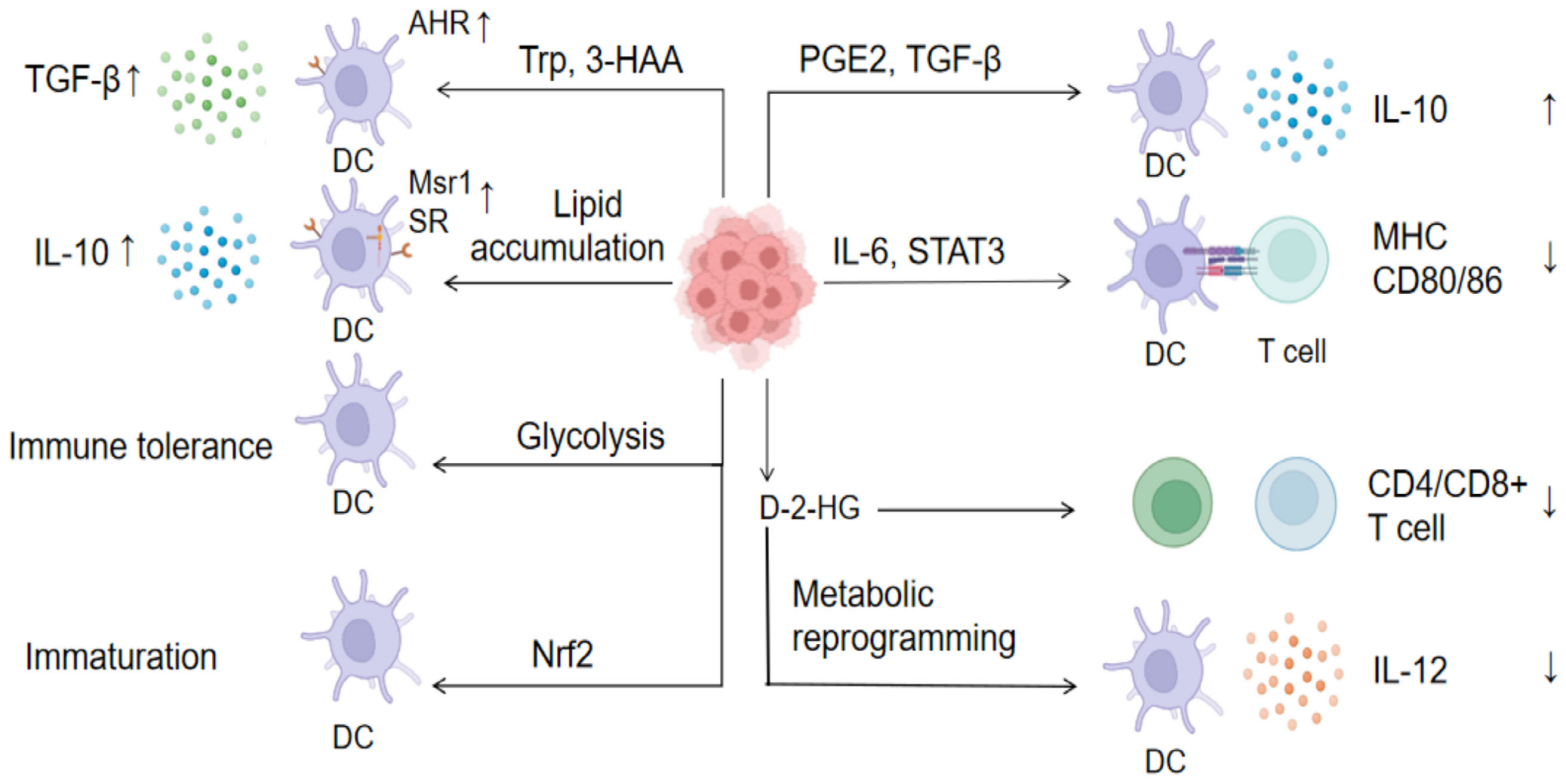
Internal resistance pathway of Glioma cells to DC function. AHR: Anti hyaluronidase reaction; DC: Dendritic cell; D‐2‐HG: D‐2‐hydroxyglutarate; FGL2: Fibrinogen‐like protein 2; HK2: hexokinase 2; IL‐6: Interleukin 6; IL‐10: Interleukin 10; IL‐12: Interleukin 12; MHC: Major histocompatibility complex; Msr1:Macrophage‐scavenging receptor 1; Nrf2: Nuclear factor erythroid 2‐ Related Factor 2; PHGDH: Phosphoglycerate dehydrogenase; SR: Scavenger receptor; STAT3: Signal transducer and activator of transcription 3; TGF‐β: Transforming growth factor‐β; Tregs: Regulatory T cells; Trp: Tryptophan; TSLP: Thymic stromal lymphopoietin; VEGF: Vascular endothelial growth factor; 3‐HAA: 3‐hydroxyamino benzoic; (1) By the secretion of inhibitory cytokines, such as IL‐10, IL‐6,TGF‐β, and VEGF. (2) By influencing the metabolic pathway, like amino acid, carbohydrate, and lipid metabolism. (3) By affecting the expression products of immunomodulatory genes like Nrf2, TSLP and D‐2‐HG.

Glioma cells can cause DCs' dysfunction in amino acid, carbohydrate, and lipid metabolism through the TME.^45^ Glioma cells can produce tryptophan, whose tryptophan metabolite 3‐hydroxyamino benzoic (3‐HAA) can enhance the transcriptional activity of AHR in NCOA7+ cDCs,^46^ thus promoting the generation of Tregs and TGF‐β. Glioma cells can also cause lipid metabolism disorders of DCs, increase the expression of macrophage‐scavenging receptor 1(Msr1) and scavenger receptor (SR) on DCs and fatty acid synthesis,^47^ cause excessive lipid accumulation in DCs, reduce their cross‐antigen presentation capacity, and produce IL‐10 to further inhibit the TME.^48^ The glucose metabolism of glioma cells can affect DC immune tolerance and malignant transformation,^49^ in which the enhanced glycolysis and increased lactate acid generation caused by hypoxia play important roles^50^ (Figure 1).

### 3.3 Suppressive effect of other cells on DCs

**FIGURE 2:**
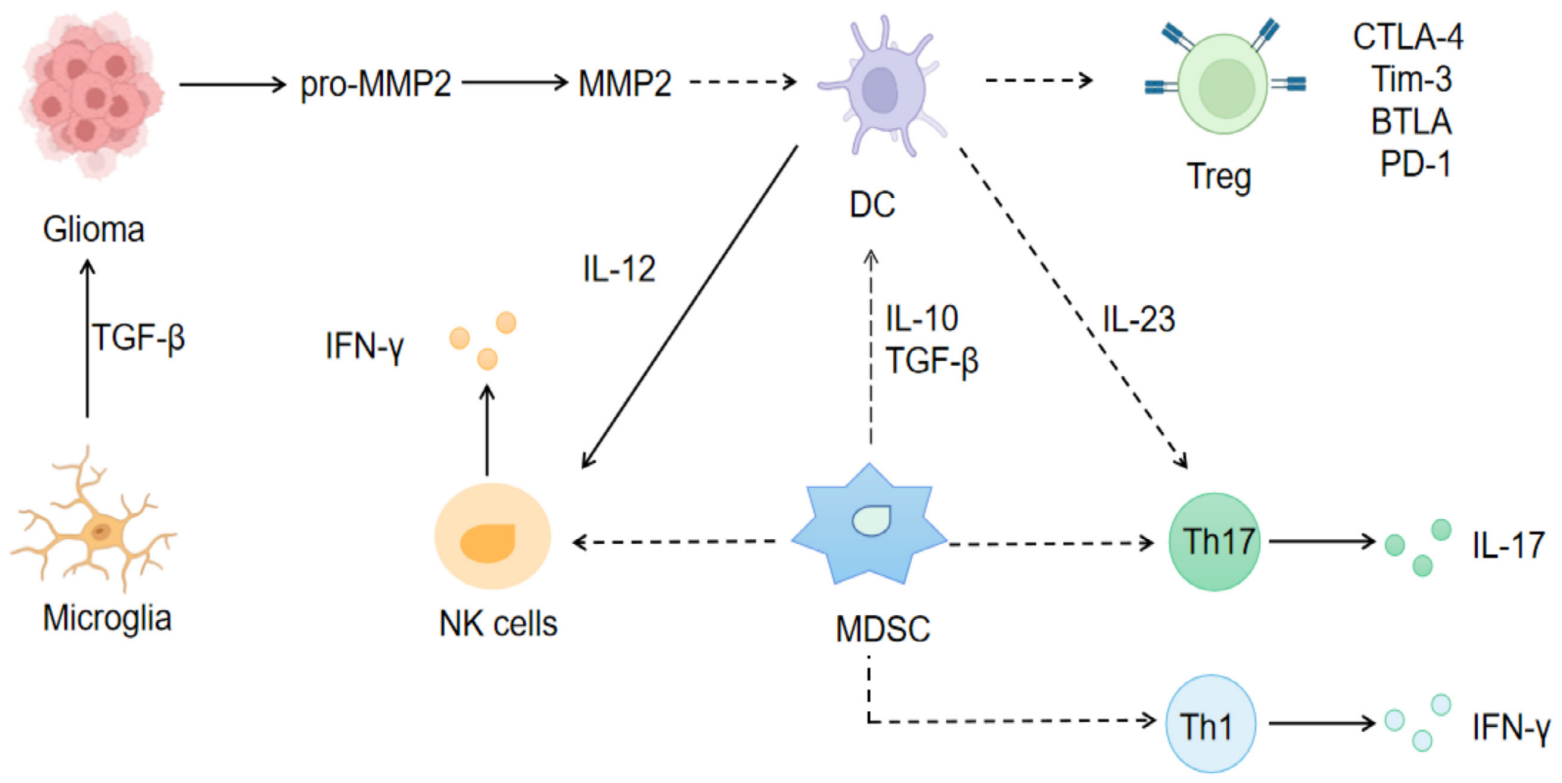
The inhibitory pathway of DC function by other immune cells. BTLA: B‐and T‐lymphocyte attenuator; CTLA‐4: Cytotoxic T lymphocyte‐associated antigen‐4; DC: Dendritic cell; GITR: Glucocorticoid‐induced tumor necrosis factor receptor; IFN‐γ: Interferon γ; IL‐10: Interleukin 10; IL‐12: Interleukin 12; IL‐23: Interleukin 23; MDSC: Myeloid‐derived suppressor cells; NK cells: Natural killer cells; PD‐1: Programmed cell death‐1; pro‐MMP2: Precursor metalloproteinase 2; TGF‐β: Transforming growth factor‐β; Tim‐3: T cell immunoglobulin domain and mucin domain‐3; The inhibitory effect of myeloid‐derived suppressor cells (MDSCs), Tregs, and microglia/tumor associated macrophages (TAMs) on DCs will damage the function of DCs and promote tumor growth and reproduction.

Microglia, myeloid‐derived suppressor cells (MDSCs), Treg cells, tumor‐associated macrophages (TAMs) and other cells interfere with the normal function of DCs to inhibit their immune response and promote glioma invasion.^51^ Microglia can upregulate recombinant sodium/hydrogen exchanger 1(NHE1) levels via colony‐stimulating factor 1(CSF‐1),^52^ release TGF‐β to trigger glioma cell production of precursor metalloproteinase 2 (pro‐MMP2), which is cleaved into active MMP2. DCs under MMP2 activation trigger the differentiation of immature CD4+ T cells into Th2 cells, thereby promoting glioma invasion.^53^ The majority of the GBM‐associated MDSCs in the mouse models are M‐MDSCs, but most MDSCs found in patient‐derived are PMN‐MDSCs.^54^ MDSCs can induce IL‐23 and Th17 generation, reduce the effects of IL‐12 and NK cells, and inhibit Th1 and IFN‐γ mediated anti‐tumor immunity, thus increasing the immunosuppression of the TME.^55^ Tregs can inhibit DC function by expressing the inhibitory receptors, such as T cell immunoglobulin domain and mucin domain‐3 (Tim‐3), B‐and T‐lymphocyte attenuator (BTLA),^56^ cytotoxic T lymphocyte‐associated antigen‐4 (CTLA‐4), and programmed cell death‐1 (PD‐1) via different mechanisms (Figure 2).

### 3.4 Inhibition of the hypoxic environment on DCs

**FIGURE 3:**
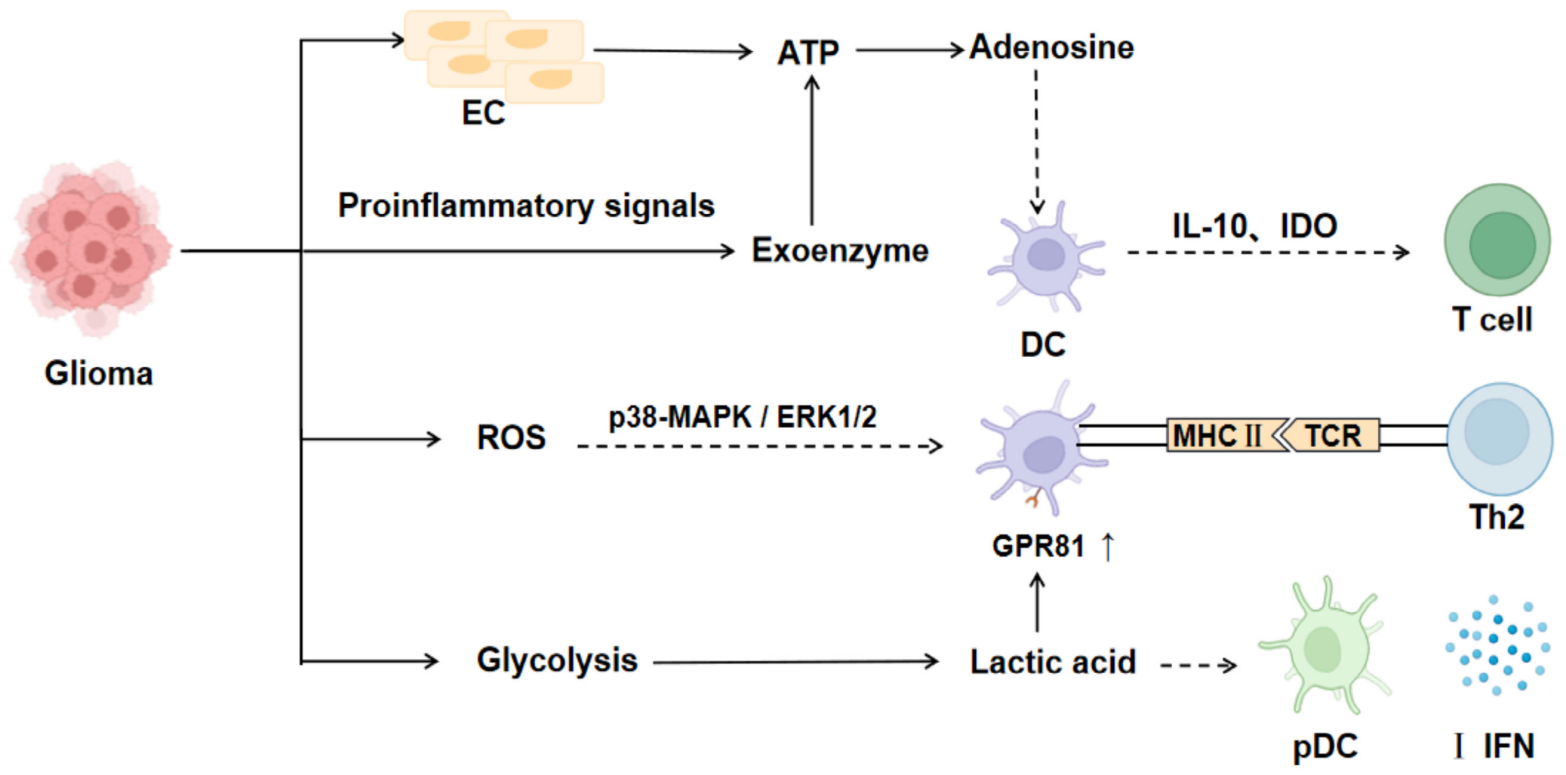
Inhibitory pathway of DC function by hypoxia. ATP: Adenosine triphosphate; EC: Epithelial cell; GPR81: G protein‐coupled receptor; IFN: Interferon; IDO: Indoleamine2,3‐dioxygenase 1; IL‐10: Interleukin 10; MHC: Major histocompatibility complex; pDC: plasmacytoid dendritic cell. Hypoxia in glioma microenvironment can easily lead to the accumulation of adenosine, the increase of lactic acid content and production of reactive oxygen, resulting in the impairment of DC function.

Glioma frequently develops in the hypoxia microenvironment, which can alter the metabolic pathways of glioma cells.^12^ The metabolic homeostasis of the brain is maintained through the interactions between its various constituent cells (such as astrocytes, neurons, and microglia).^57^ However, this balance can be altered by genomic aberrations and biochemical changes.^58^ On the one hand, hypoxia can disrupt the BBB through HIF‐1 mediation, thus initiating astrocytes and pericytes to resist hypoxia.^21^ Astrocytes maintain ATP production mainly through the upregulation of glycolytic enzymes and angiogenesis factor genes.^59^ In addition, both astrocytes and pericytes can combat the damage from hypoxia by producing large amounts of VEGF and MMP9, which also interferes with DC maturation.^60^ On the other hand, glioma cells generate pro‐inflammatory signals in response to hypoxic stress,^61^ triggering the active release of ATP through junctin and total junctin channels expressed by endothelial cells.^59^ Glioma cells also promote the excessive accumulation of adenosine in the TME by producing extracellular enzymes that convert ATP into adenosine,^62^, ^63^ thus interfering with the function of DCs, allowing them to produce large amounts of immunosuppressive factors and upregulate IDO expression.^64^ Hypoxia produces a large amount of lactate and enhances glycolysis functions.^65^ The elevated lactate not only activates the G protein‐coupled receptor GPR81 on DCs, which promotes the growth of tumor cells and thus inhibiting MHCs on DCs,^66^ but also inhibits the release of IFN‐α and IFN‐γ from pDCs through this receptor, and weakening the anti‐tumor immunity caused by DCs.^67^ Moreover, the elevation of ROS can cause T cell dysfunction by affecting the p38‐MAPK and ERK1/2 pathways to inhibit DC maturation and antigen‐presenting function^34^, ^67^ (Figure 3).

## 4 INFLUENCE OF DC VACCINES ON THE IMMUNE MICROENVIRONMENT OF GLIOMA PATIENTS

In glioma patients, DCs are maintained with low function or tolerance due to the inhibitory effect of immune microenvironment on DC proliferation and differentiation. Therefore, by injecting active DCs that mature in vitro, DCs can activate inhibited T cells undergoing lymphatic reflux into the brain, thus playing a relative compensatory role and enhancing the adaptive immune response in patients.

### 4.1 Cultivating mature DCs is a prerequisite for improving the microenvironment

**FIGURE 4:**
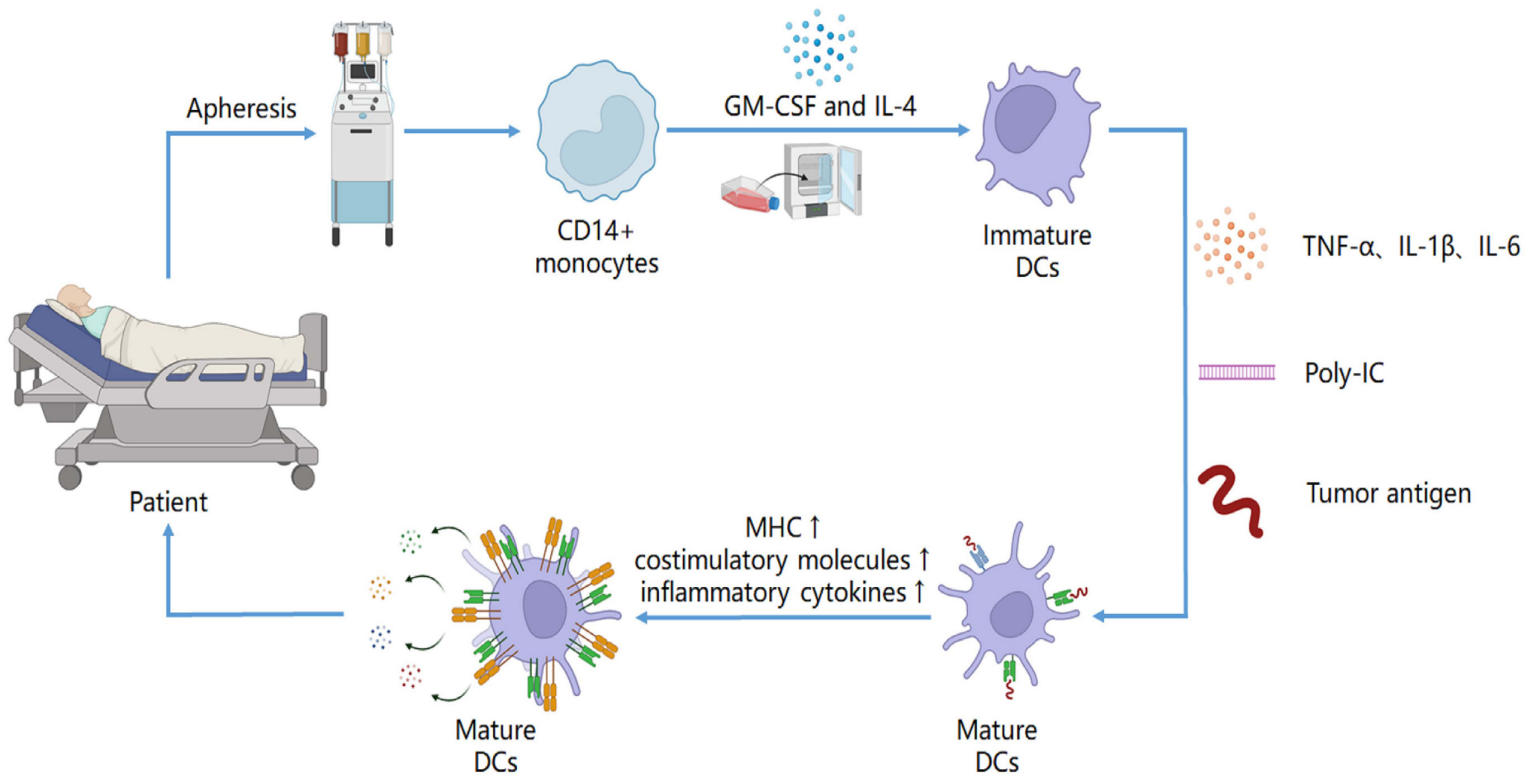
Mature DC were cultured to improve the patient's tumor microenvironment. DC: Dendritic cell; IL‐1β: Interleukin 1β; GM‐CSF: Granulocyte macrophage colony stimulating factor; IL‐4: Interleukin 4; IL‐6: Interleukin 6; poly‐IC: Polyinosinic polycytidylic acid; TGF‐α: Transforming growth factor‐α. DC maturation is stimulated by further cocktail therapy to induce high expression of MHC and positive costimulatory molecules, promoting the secretion of inflammatory cytokines and chemokines, and ultimately enhancing the immune response mediated by T cells.

Low‐function DCs highly express antigen uptake receptors and show low expression of co‐stimulatory molecules and chemokines.^68^ DC vaccines currently use cocktail therapy to cultivate mature DCs. CD14+ monocytes are initially isolated from peripheral blood mononuclear cells (PBMCs) in the patient and mix them with GM‐CSF and IL‐4 for 5–7 days to convert the monocytes into immature DC cells.^69^ Subsequently, the DCs are mixed with IL‐1β, IL‐6, TNF‐α for 16 to 20 hours and pulse them with tumor antigen to allow antigen uptake and presentation by the DCs.^70^ DC maturation is stimulated by further cocktail therapy to induce high expression of MHCs and positive costimulatory molecules (e.g., CD80/86), promote the secretion of inflammatory cytokines (e.g., IL‐12, TNF‐α) and chemokines (e.g., CXCL9/10),^71^ and ultimately enhance the immune response mediated by T cells and immune cells migrate to the tumor site, thus improving the tumor microenvironment^72^ (Figure 4).

In 2001, Kikuchi et al. vaccinated glioma patients through fusion cells of DC and tumor cells from polyethylene glycol, this early exploration of clinical trials proved that DC vaccines can improve patients' immune response. In 2004, S Rutkowski et al stimulated DC maturation with GM‐CSF, IL‐4, and PGE2 and pulsed with glioma cell lysates supernatants undergoing six freeze–thaw cycles, resulting in two out of six patients with complete resection a median overall survival greater than 35 months. With the continuous exploration of the genome, the preparation of mature DC has been further improved. In terms of tumor antigens used for pulse, glioma‐associated antigens (GAA) can be selected, such as WT1, TRP2, and IL‐13Rα2, or glioma‐specific antigen (GSA) EGFRvIII, while different antigen stimuli have discrepancy effects on DC function. Robert M. Prins et al. compared the efficacy of DCs loaded with tumor‐associated antigen peptides and tumor lysates, indicating that NK cells in patients loaded GAA have a longer survival in the loaded tumor lysates, this possibly because PGE2 exists in the cytokine mixture added in the GAA preparation, which has been shown to promote Treg cell proliferation, reducing the immune response. Other clinical trials of the new methods are still ongoing (Table 1).

**TABLE 1:** Clinical trials of glioma DC vaccines from the perspective of cultured mature DC DTH: Delayed type hypersensitivity; GAA: Glioma associate antigen; GM‐CSF: Granulocyte‐macrophage colony‐stimulating factor; IFN‐α: Interferon‐α; IFN‐γ: Interferon‐γ; IL‐4: Interleukin‐4; IL‐6: Interleukin‐6; IL‐1β: Interleukin‐1β; OS: Overall Survival; PFS: Progression‐free survival; PGE‐2:Prostaglandin E2; poly‐IC: Polyinosinic‐ Polycytidylic acid; TNF‐α: Tumor necrosis factor‐α; TTP: Time to progression. ICT‐107^↑^: DC pulsed with MAGE‐1, HER‐2, AIM‐2, TRP‐2, gp100, and IL‐13Rα2

Clinical trial	Year	Phase	Condition	Antigen	A mixture of cytokines that induce DC maturation	Survival time	Immunological reaction	References
Yu et al.	2001	I	Glioblastoma, anaplastic astrocytoma	Autologous tumor peptide	GM‐CSF+ IL‐4	OS: 455 days	Two of the four patients developed a strong CD8+ T and CD45RO+ memory T cell infiltration in the tumor region	^122^
Kikuchi et al.	2001	I	Recurrent malignant glioma	Tumor cells were fused with the DC	GM‐CSF+ IL‐4+ TNF‐α		The percentages of CD16+ and CD56+ lymphocytes increased slightly in the peripheral blood, and the IFN‐γ concentration in the supernatant increased	^123^
Rutkowski et al.	2004	I	Recurrent malignant glioma	Tumor lysates	GM‐CSF, IL‐4, PGE2	Two out of six patients had a median overall survival greater than 35 months	Six out of eight of the patients receiving the DTH experiment were positive	^124^
Yamanaka et al.	2005	I/II	Recurrent malignant glioma, III Recurrent malignant glioma, IV	Tumor lysates	GM‐CSF+ IL‐4	OS: 480 days	Initiates a specific T‐cell response	^125^
Okada et al.	2011	I/II	Glioblastoma, Anaplastic astrocytoma, Anaplastic Oligodendroglioma, Anaplastic Oligoastrocytoma	EphA2 IL‐13Rα2 YKL‐40 gp100	TNF‐α, IFN‐α, IL‐1β, IFN‐γ, poly‐IC	TTP: 4 months	αDC1 produces IL‐12 and induces an epitope‐specific immune response against GAA, and IFN‐γ upregulation might be related to the induction of an adaptive immune response	^126^
Prins et al.	2013	I	Glioblastoma	TRP2, gp100, her2/neu	TNF‐α, IL‐6, IL‐1β, PGE2	OS: 14.5 months	Reduced post‐vaccination/pre‐vaccine Treg ratio and reduced frequency of activated NK cells were associated with prolonged patient survival	^80^
Sakai et al.	2015	I	Glioblastoma, anaplastic astrocytoma, Anaplastic Oligoastrocytoma, Oligodendroglioma	WT‐1	Saphlin OK‐432, PGE2, IL‐4, GM‐CSF	OS: 26 months	Demonstrated that prolonged survival was associated with increased T lymphocytes	^127^
Liau LM et al.	2018	III	Glioblastoma	Tumor lysates	—	Vaccine group OS: 23.1 months, methylated MGMT group OS: 34.7 months	—	^128^
Wen PY et al.	2019	II	Glioblastoma	ICT‐107^↑^	GM‐CSF, IL‐4, IFN‐γ	OS: 17.0 months PFS: 11.2 months	Systemic cytokine‐response IFN‐γ and TNF‐α occurred in 33% of the patients and were associated with a trend toward improved survival	^129^
NCT01567202	2012	II	Glioma, Glioblastoma Multiforme Neoplasms	Glioma stem cells	Recruiting			^130^
NCT02010606	2013	I	Glioblastoma, Glioblastoma Multiforme, Glioma, Astrocytoma, Brain Tumor	Glioma stem cells	Completed, no results released			^130^
NCT02287428	2014	I	Glioblastoma	Personalized neoantigens	Recruiting			^130^
NCT02465268	2015	II	Glioblastoma Multiforme, Glioblastoma Malignant, Glioma, Astrocytoma, IV Glioblastoma	CMV pp65‐LAMP mRNA	Recruiting			^130^
NCT02366728	2015	II	Glioblastoma, Astrocytoma, IV Giant Cell glioblastoma, glioblastoma multiforme	CMV pp65‐LAMP mRNA	Completed			^130^
NCT02709616	2016	I	Glioblastoma	Personalized neoantigens	Completed			^130^
NCT02649582	2016	I/II	Glioblastoma	WT1 mRNA	Recruiting			^130^
NCT03914768	2019	I	Diffuse Intrinsic Pontine Glioma, Glioblastoma	Tumor cells or tumor‐associated antigens	Enrolling by invitation			^130^
NCT04277221	2020	III	Glioblastoma Multiforme	Autologous Dendritic Cell/Tumor Antigen, ADCTA	Recruiting			^130^
NCT04968366	2021	I	Glioblastoma Multiforme of the Brain	Tumor neoantigen peptide	Recruiting			^130^
NCT05100641	2021	III	Glioblastoma	Autologous tumor antigen‐pulsed DC vaccine (AV‐GBM‐1)	Not yet recruiting			^130^

### 4.2 Improving the TME by regulating the expression of MHCs and co‐stimulators

Mature DC can exert its therapeutic effect by upregulating stimulant receptors (CD80/86, etc.) or reducing inhibitory receptors (PD‐L1, CTLA‐4, etc.). Among them, CTLA‐4 and PD‐L1 are often used as immunodetection indicators for patients with glioma after treatment.^73^ Chia‐Ing Jan et al treated 27 tumor antigen‐DC patients with GBM and found patients whose tumor‐infiltrating lymphocytes (TIL) with a lower PD‐1+/CD8+ ratio (>0.21) have a longer OS and PFS (median P S 60.97 months, *p* < 0.001 and PFS 11.08 months) (*p* < 0.008 months).^74^ As the efficiency of cytotoxic T cells killing of tumor cells upon reaching the tumor microenvironment depends on the proportion of PD‐1+ cytotoxic T cells,^46^ the vaccination of DC vaccines can significantly reduce T cell expression of PD‐1, thus improving the tumor microenvironment through the above pathway.^75^

During the preparation of DC vaccines, immature DCs can be exposed to mature signals via stimulation with certain medicine to obtain a mature phenotype, which further upregulates positive costimulatory molecules such as CD40, CD86 and OX40L.^76^ These stimulatory drugs include a TriMix (a mixture of TLR4, CD40 and CD70),^77^ a Toll‐like receptor (TLR) agonist, tetanus toxoid, Flt3L and STING.^78^ One of the most common protein is TLR, which activates the MAPK and NF‐κB pathways to induce multiple costimulatory molecules,^79^ CCR7 and pro‐inflammatory cytokines to promote multiple inflammatory cascades, thus enhancing the body's immune response.^69^ In the glioma patients vaccinated with imiquimod adjuvant DC pulsed by tumor lysate, Robert M. Prins et al. found that the median overall survival in patients newly diagnosed and receiving DC vaccines was significantly higher at 35.9 months than before treatment (overall survival was 15.9 months).^80^ The number of CD3+ and CD8+ TIL combined with DC increased significantly and was associated with clinical outcomes, thereby improving the tumor microenvironment and median survival.^69^ CD40 is a member of the TNF receptor superfamily on APC, which can enhance the expression of MHCs,^81^ the production of costimulatory molecules, and the interaction between T cells and DCs to improve the tumor microenvironment.^78^ In addition, the combination of different immune stimulators might affect their anti‐tumor immune response differently.^82^ The combination of immune stimulators of CD40 and TLR highly inhibited the tumor growth in mice,^83^ whereas the combination of TLR7 with an activator of TLR9 reduces NF‐κB activation and compromises vaccine efficacy.^84^ The use of imiquimod, based on GM‐CSF, also results in an increase in MDSCs and Tregs. Therefore, we should choose the combination of different stimulators carefully.

### 4.3 Improving the TME by regulating cytokines

After pulsing through tumor antigens, the DCs regulate the expression of proinflammatory cytokines, reduce negative cytokines, and regulate the migration of other immune cells,^85^ thus enhancing the anti‐tumor immunity of the body and improving the tumor microenvironment.^86^ A study showed that intra tumoral (IT) injection of antigen‐pulse DC cells improves the TME by reducing TGF‐β,^87^ increasing TNF‐α and IFN‐γ, promoting proliferations of CD8+ T cells, reducing Tregs activation, and increasing the survival rate of mice with glioma.^88^ Moreover, it was shown in clinical trials that DC vaccines can significantly increase the patient serum levels of NK cells, IL‐2 and IL‐12, reduce the levels of CD133+ tumor stem cells to improve the microenvironment, and this is associated with improved survival.^89^ Nine months after vaccination, the tumor control rate and patient survival rate improved significantly compared with the control group (*p* < 0.05), while the time to relapse was significantly longer than the control group (*p* < 0.05).^90^ However, the rise in IFN‐γ only occurred after the first vaccine, indicating that IFN‐γ may improve patients' immune microenvironment by inducing an adaptive immune response.^8^ DC vaccines pulsed by the cocktail method are more obvious for IFN‐γ and IL‐12 mediated T cell activation, which illustrates an important role of IFN‐γ in DC vaccine treatment.^91^

### 4.4 Improve the TME by regulating immune cell migration

Mature DC cultured in vitro can induce other immune cells to migrate to the tumor site by chemokines, thus improving the immune microenvironment of patients.^92^ The mRNA levels of both CCL10 and TLR3 were significantly upregulated after the first and fourth DC vaccination, and CCL10 could guide CD8+ T cells into brain tumor sites,^93^ thus improving the inhibitory immune microenvironment in glioma patients.^94^ Hirokazu Ogino et al. pretreated DCs with poly‐ICLC and found that in addition to upregulating cytokines such as IFN‐γ, TNF‐α, and IL‐10,^95^ the migration of effector memory CD8+ T cells in the TME may be mediated through the CXCL10/CXCR3 axis,^96^ which showed the activating DCs can effectively improve the migration of other immune cells and improve the tumor microenvironment by regionalization factors. In addition, other studies had applied Td and TNF‐α to promote DC migration in lymph nodes. Td pretreatment for 4–6 h before DC vaccination followed by DC vaccination with albumin RNA showed a 3‐fold increase in inguinal lymph node afferent DCs,^97^ possibly caused by the Td recall response and increased CCL3 levels, which is associated with prolonged patient survival, thus improving immune function by promoting DC migration into lymph nodes.^98^

## 5 EXISTING CHALLENGES AND FUTURE APPROACHES TO DC IMMUNOTHERAPY

Although clinical trials have demonstrated that DC vaccines can improve the glioma immune microenvironment and prolong patient survival,^99^ some of them did not entry phase III or improve recurrent GBM patients probably because of the limited ability of DC vaccines to improve the powerful inhibitory microenvironment of glioma.^100^ Therefore, we could further optimize DC vaccines from perspectives of improving the microenvironment.

### 5.1 Challenge and Methods 1: Standardizing DC maturation methods

There are marked differences in the maturation processes and methods of DCs in different clinical trials, and we lack mature processes to guide them. Commonly used cytokine mixtures for maturation include IL‐4, GM‐CSF, IL‐1β, IL‐3, IL‐6, TNF‐α, and IFN‐γ,^101^ and some also add PGE2, which has been shown to cause proliferation of Tregs.^102^ Differences in HLA typing between patients mean that the T cell response caused by mature DCs prepared by different methods are also different. Therefore, formulating a standardized DC preparation process will be conducive to produce a better treatment effect.^103^

### 5.2 Challenges and Methods 2: Monocyte‐derived dendritic cells (MoDCs) have limited function—exploring cDCs, pDCs, and exosome‐based vaccines

Most vaccine experiments have been performed through MoDCs. However, the in vitro culture is functionally different from native MoDC growth in vivo.^104^ Long‐term culture might lead to decreased migration capacity and loss of function, and it is associated with T lymphocyte depletion.^105^ Therefore, MoDCs may not be the most appropriate DC cell subtype for vaccine preparation.^106^ In the future, vaccines based on natural circulating DCs, such as cDCs, pDCs, or exosomes should be explored to achieve improved results.^107^ Actually, the cDCs have been shown to elicit a stronger CD8+ T cell response than pDCs.^108^

### 5.3 Challenges and Methods 3: Limited loading methods of tumor antigens‐‐ Optimizing loading methods for tumor antigens

Different loading methods of tumor antigens can lead to discrepancies in treatment efficacy. At present, the commonly used tumor antigens include tumor lysates, peptides,^109^ nucleic acids and neoantigen, etc. Tumor lysates contain varieties of tumor antigens and unique neoantigens,^110^ but other unrelated antigens existing in the lysate may dilute specific immunogenic antigen, thus reducing the antigen uptake and presentation of DCs.^111^ Peptides are widely applied in the clinical trials, including GAA‐derived peptides and GSA‐derived peptides.^102^ However, peptide‐pulsed DC vaccines may activate some effector T cells that are not expressed yet, interfering with the activity of other anti‐tumor T cells, so it still need to be further explored. Besides the common electroporation methods,^112^ mRNA can also be loaded with lipid nanoparticles (LNP). Nanocarriers is able to effectively prevent RNA degradation and increase its stability,^113^ simultaneously package the immune adjuvant to increase the immunogenicity of the vaccine, increase the cross‐presentation of antigen, and induce DC maturation and increase the CTL response.^114^ Therefore, the use of LNP can shorten the time needed to produce a personalized vaccine, and extend the shelf life of the vaccine, which has a relatively broad development prospect.^115^

As for the new technique of personalized antigens,^97^ patient tumor individualized sequencing, analysis, identification, and screening are time‐consuming process.^116^ Therefore, new antigen sequencing and screening technology should be developed to further promote its wider application in the future,^117^ such as using full exon sequencing technology, high throughput sequencing screening and identification, and choosing automated, rather than manual, super high efficiency liquid chromatography (UPLC).^118^

### 5.4 Challenges and Method 4: Single use of a DC vaccine with limited efficacy — using combinatorial therapy

Targeting multiple pathways through DC vaccines combined with other therapies might be an important method to combat immunosuppression in the TME. Currently, the treatment of GBM comprises firstly using surgical resection to reduce the tumor load and prolong the survival time.^119^ Then combine DC vaccines with radiotherapy, chemotherapy, or both to induce DNA damage and endoplasmic reticulum stress to stimulate cell death, release chemokines and cytokines to increase the DC stimulation signals, thus supplementing the effect of anti‐tumor DC vaccines. We can also combine specific targeted therapy to block the pathways besides activating DC, such as targeting the BBB to increase drug delivery, targeting signaling pathways such as p53, RTK and Rb,^94^ or cytokines to specifically block MDSCs, Tregs and microglia. For example, BLZ945 can block CSF‐1R to reduce the activity of microglia and the activation of M2 macrophages,^120^ thereby enhancing the body's immune response and the median survival. If combined with DC vaccines, it would be helpful to decrease tumor cell immune evasion and provide new directions to prolong median survival.^121^

## 6 CONCLUSIONS

DC vaccines can upregulate the expression of MHCs and co‐stimulators, and promote the secretion of cytokines and chemokines, thus increasing the number of activated effector T cells and promoting the migration of immune cells to improve the immunosuppressive microenvironment of glioma patients. It will bring good treatment prospects to patients. Although existing studies have shown that DC vaccines have a role in improving the tumor microenvironment, such effects are not entirely consistent with the improvement in clinical outcomes of patients. Possible reasons for this result are imperfect immune detection endpoints and the lack of corresponding administration standards, etc. Some studies also show that the age of GBM patients may also be a reason as they found that the use of DC vaccines in the GBM population younger than ordinary patients can show some correlations. Therefore, if further studies can overcome above deficiencies, DC vaccines will have promising development prospects.

## AUTHOR CONTRIBUTIONS

**Jing Zhou:** Writing – original draft (lead); writing – review and editing (lead). **Luohong Li:** Conceptualization (equal); writing – review and editing (equal). **Minqi Jia:** Software (equal); writing – review and editing (equal). **Qianjin Liao:** Data curation (equal). **Guiping Peng:** Formal analysis (equal). **Gengqiu Luo:** Data curation (equal). **Yanhong Zhou:** Data curation (equal).

## CONFLICT OF INTEREST

The authors declare that the research was conducted in the absence of any commercial or financial relationships that could be construed as a potential conflict of interest.

## ETHICS STATEMENT

Ethical approval statement is not applicable to this article.

## Data Availability

Data sharing not applicable to this article as no datasets were generated or analyzed during the current study.
